# Sexual Response Problems and Their Correlates Among Older Adults From the Sexual Well-Being (SWELL) Study in China: Multicenter Cross-Sectional Study

**DOI:** 10.2196/66772

**Published:** 2025-05-01

**Authors:** Bingyu Liang, Chen Xu, Bingyi Wang, Xinyi Li, Xin Peng, Ying Wang, Hui Li, Yong Lu, Xiaopei Shen, Lin Ouyang, Guohui Wu, Maohe Yu, Jiewei Liu, Xiaojun Meng, Yong Cai, Huachun Zou

**Affiliations:** 1 School of Public Health, Guangxi Key Laboratory of AIDS Prevention and Treatment, Guangxi Medical University, Nanning, China; 2 Department of Clinical Research, Faculty of Infectious and Tropical Diseases, London School of Hygiene and Tropical Medicine, London, United Kingdom; 3 Public Health Research Center, Hongqiao International Institute of Medicine, Tongren Hospital, Shanghai Jiao Tong University School of Medicine, Shanghai, China; 4 School of Public Health (Shenzhen), Sun Yat-sen University, Shenzhen, China; 5 School of Public Health, Shanghai Jiao Tong University School of Medicine, Shanghai, China; 6 Department of AIDS/STD Control and Prevention, Shizhong District Center for Disease Control and Prevention, Jinan, China; 7 School of Public Health, the Key Laboratory of Environmental Pollution Monitoring and Disease Control, Guizhou Medical University, Guizhou, China; 8 Department of AIDS/STD Control and Prevention, Affiliation Shanghai Municipal Center for Disease Control and Prevention, Shanghai, China; 9 Department of AIDS/STD Control and Prevention, Chongqing Municipal Center for Disease Control and Prevention, Chongqing, China; 10 Department of AIDS/STD Control and Prevention, Tianjin Center for Disease Control and Prevention, Tianjin, China; 11 Baiyun District Center for Disease Control and Prevention, Guanghzou, China; 12 Department of AIDS/STD Control and Prevention, Wuxi Municipal Center for Disease Control and Prevention, Wuxi, China; 13 School of Public Health, Fudan University, 130 Andong Road, Xuhui District, Shanghai, 200032, China, 86 13926420419

**Keywords:** dysfunction, sexual health, sexual well-being, sexually active, sexual activity, well-being, correlate, sex partner relationship, gerontology, geriatrics, older adults, elder, elderly, older person, aging, China, cross-sectional study

## Abstract

**Background:**

Sexual response problems among older adults are not an inevitable consequence of aging but rather a response to sexual health. However, there is a lack of recent and multicenter data on this issue in China.

**Objective:**

This study aims to assess the prevalence of sexual response problems and their correlates among older adults.

**Methods:**

A multicenter cross-sectional study on sexual well-being was conducted among individuals aged more than 50 years in China between June 2020 and December 2022. Data on sociodemographics, physical health, psychological health, and sexual response problems were collected through face-to-face interviews. We included sexually active older adults who reported either vaginal, oral, or anal sex in the past 12 months for this study. Sexual response problems included a lack of interest or enjoyment in sex; feeling anxious, having pain, or no excitement during sex; no desire or orgasms; and the lack of lubrication in sex. The stepwise logistic regression models were used to examine the correlates of sexual response problems.

**Results:**

A total of 1317 sexually active older adults (842 men, 475 women) were included. Older women reported a higher prevalence of sexual response problems than older men (52.0% [247/475] vs 43.1% [363/842]). Common factors associated with at least one of the sexual response problems included living in rural areas (men: adjusted odds ratio [aOR]=0.31, 95% CI 0.22‐0.43; women: aOR=0.29, 95% CI 0.19‐0.43) and abnormal BMI (aOR=men: 1.52, 95% CI1.11‐2.07; women: aOR=2.19, 95% CI 1.47‐3.28). Among older men, sleep quality (aOR=1.87, 95% CI 1.30‐2.68), emotional connection with sex partners during sexual intercourse (aOR=0.69, 95% CI 0.50‐0.96), frequently experienced fatigue (aOR=2.47, 95% CI 1.59‐3.90), anxiety (aOR=4.26, 95% CI 1.12‐21.27), and seeking professional help for sex life (aOR=1.58, 95% CI 1.14‐2.21) were associated with sexual response problems. Among older women, sexual response problems were associated with a lack of physical exercise (aOR=1.69, 95% CI 1.13‐2.54), poor sex-partner relationships (aOR=1.70, 95% CI 1.12‐2.60), and depressive symptoms (aOR=3.18, 95% CI 1.18‐10.24).

**Conclusions:**

Sexual response problems are common among older adults. These problems were associated with adverse physical health, mental health, and poor sex-partner relationships. These findings highlight the importance for health care providers to take into account the physical and psychological health of older adults, as well as the quality of their relationships with sexual partners when diagnosing and addressing sexual response problems.

## Introduction

Sexual response problems are characterized by diminished or absent sexual interest and disruptions in the physiological or psychosocial patterns associated with the sexual response cycle. These disruptions include a lack of interest, enjoyment, desire, orgasms, and lubrication in sex [1]. Sexual response problems have a profound impact on various aspects of life for older adults, affecting their quality of life, interpersonal relationships, dissatisfaction in marriage [2,3], work efficiency [4], self-esteem [5], and physical and mental health [2,3,6].

Recent studies have shown a high prevalence of sexual problems in both older men and older women. For instance, in Europe, a majority of men (73.7%‐79.8%) and women (23.5%‐50.2%) reported experiencing at least one sexual problem, with the most prevalent sexual problems being orgasmic difficulties and reaching orgasm more quickly than desired and failure to reach orgasm or taking too long to climax [7,8]. An early national survey in urban China indicated that 21% of men and 35% of women aged 20‐64 years had at least one persistent sexual dysfunction, with lack of sex interest, erectile difficulties (men), and inability to reach orgasm being the most common problems [9]. There are substantial variations in the prevalence of sexual response problems across different global regions, with noticeable differences between men and women. As extensively demonstrated by a significant number of epidemiological studies worldwide, there are substantial differences in the prevalence of sexual response problems among men and women. A few researchers have suggested that the prevalence of sexual response problems was higher in men compared to women [10,11]. Conversely, more studies have reported a higher prevalence among females [12-15]. It is necessary to elucidate the specific causes of these differences and inform gender-specific prevention and intervention strategies.

Sociodemographic, psychological, sex partner relationships, chronic disease, and physical health were found to be important determinants of sexual response problems among older adults. The prevalence of sexual problems tends to increase with age [16], and there are notable differences between men and women in terms of prevalence and types of sexual response problems [17]. Psychosocial factors such as anxiety, depression, stress, and the quality of marital relationships play significant roles in sexual response problems [16,18,19]. Intimate partner violence is associated with a higher likelihood of experiencing sexual problems [20]. In addition, chronic diseases like cancer [17], diabetes [21], and coronary heart disease [22] are linked to an increased risk of sexual dysfunction. Moreover, sexual response problems in one partner may influence the sexual function of the other partner [23].

Despite extensive studies on sexual problems, most of the existing literature was from developed countries or early studies, and there is a lack of nationally representative, large sample and recent data on the prevalence and correlates of sexual response problems among older adults in China. Given China’s rapidly aging population [24,25], the sexual health of older adults is a growing concern. A comprehensive understanding of older adults’ sexual response problems may enhance sex education, research, policy, and clinical care for this growing population. This multicentre cross-sectional study, using data from the Sexual Well-being (SWELL) study in China, aims to fill the research gap by examining the prevalence of sexual response problems and their correlates among older adults. These epidemiological data are essential for andrologists, gynecologists, urologists, venereologists, and other health care providers involved in treating and caring for older adults. They may help them counsel their patients on the potential adverse effects of different treatment modalities. Moreover, the findings are expected to contribute valuable insights for developing targeted interventions to enhance sexual relationships, improve quality of life, and address the sexual health challenges faced by aging populations in China.

## Methods

### Study Participants and Procedures

Our study was based on the SWELL study, a multicenter cross-sectional survey conducted between June 2020 and December 2022. The survey spanned four different regions in China, including Shanghai (Eastern China), Jinan (Eastern China), Chongqing (Western China), Guangzhou (Southern China), and Tianjin (Northern China). Participants were recruited using a multistage sampling design, and more detailed sampling procedures are provided in our previous protocol [26]. We collected data on demographic characteristics, physical health characteristics, mental health characteristics, sex partner relationship characteristics, and sexual behavior characteristics through face-to-face interviews. All participants provide formal informed consent to participate in the study.

Participants were enlisted from subdistricts within each chosen city. Eligibility criteria for participants in this study included: (1) aged 50 years and older; (2) only heterosexual orientation; (3) having engaged in heterosexual activities (including oral or vaginal intercourse) in the preceding year; (4) being able to comprehend the survey instrument of the SWELL Study.

### Ethical Consideration

The SWELL study was approved by the School of Public Health (Shenzhen), Sun Yat-sen University Research Ethics Committee (approval number SYSU-PHS [2019] 006) and was performed following the Helsinki Declaration. Written consent was obtained from all participants, who were informed of their right to withdraw from the study at any time. Participant information and responses remained confidential, with anonymized data stored in password-protected folders accessible exclusively to the research team and supervisors.

### Study Variables

#### Sexual Response Problems (Outcomes Variables)

Respondents were asked to report if they had experienced any of the following sexual response problems for three months in the preceding year: (1) lacked desire for sex, (2) lacked enjoyment in sex, (3) anxiety during sex, (4) discomfort or pain in sex, (5) no sexual arousal or excitement during sex, (6) lack of or delayed orgasm despite arousal, and (7) reaching orgasm faster than you would like, (8) lubrication difficulties (women only) or erectile function difficulties (men only). These items captured the major sexual response problem domains in the classification of sexual dysfunction in the *Diagnostic and Statistical Manual of Mental Disorders*, Fourth Edition [27]. The reliability of sexual response problems in this study was 0.75.

#### Sociodemographic Characteristics

Age, gender (men and women), living area (rural or urban), monthly income (Chinese Yuan [CNY]), and years of education (<6 years: primary and lower; 7‐12 years: senior or high school; >12 years: college and above) were included in demographic characteristics. Age was categorized into 3 age groups (50‐59 years, 60‐69 years, and older than 70 years). Monthly income (CNY) was categorized into 2 groups (≥5000 CNY [US $700] and <5000 CNY [US $700]).

### Lifestyle Characteristics

#### Physical Exercise

Physical exercise was assessed with 5 items that inquired about the frequency of participation (6 times a week, 3‐5 times a week, 1‐2 times a week, no more than once a week, hardly ever, or never). The participants who reported engaging in physical exercise more than 1‐2 times a week were categorized as often engaging in physical exercise. The remaining responses were categorized as not often exercised [28].

#### Seeking Professional Help for Sex Life

Participants were asked whether they had sought help or advice about their sex life from a range of sources in the past year. These sources included 4 informal sources (family member or friend, information and support sites on the internet) and 6 professional sources (general practitioner or family doctor, sexual health clinic, genitourinary clinic, sexually transmitted infection clinic, or relationship counselor); more than one answer was allowed. If a participant responds that they have previously sought help for sex life from 6 professional sources, this is defined as “seeking professional help for sex life.”

### Physical Health Characteristics

#### Frequently Experiencing Fatigue

As for frequently experiencing fatigue, the participants were asked about their fatigue levels using a question: “Do you frequently experience fatigue?” with response options of “Yes” or “No.”

#### Chronic Disease

Chronic disease is defined as one or more diseases that last for 3 months or more including cardiovascular diseases (including myocardial infarction, coronary heart disease, angina, other forms of heart disease, and hypertension), arthritis, diabetes, or hyperglycemia, cerebral hemorrhage or stroke, chronic lung disease (excluding asthma), Parkinson disease, epilepsy, hyperlipidemia, gout or hyperuric acid, chronic gastroenteritis and chronic pain. A separate section was directed at women participants, whether they have a history of one of the following, including bladder surgery, genital or gynecologic surgery, cesarean section, abdominal surgery, and hip pelvic fractures or hip replacement.

#### Body Mass Index and Sleep Quality

In the SWELL Study, BMI is considered abnormal when lower than 18.5 or higher than 25.

Sleep quality was evaluated using the validated single-item sleep quality scale (SQS), which ranges from 1 to 10 and has been proven to be divided into 2 categories for analysis: those indicating poor sleep quality (scores 1‐6) and those indicating good sleep quality (scores 7‐10) [29]. The SQS had an acceptable internal consistency (Cronbach α=0.85).

### Mental Health Characteristics

#### Depressive Symptoms

Depressive symptoms were measured by the 9-item Patient Health Questionnaire (PHQ-9), which has been validated and proven [29,30]. The scale’s total score ranges from 0 to 27, with scores≥10 representing clinically significant depressive symptoms. This study defined a score greater than or equal to 10 as depressive symptoms.

#### Anxiety Symptoms

The anxiety symptoms were measured on the generalized anxiety disorder-7 (GAD-7) scale with 7-item. Mild or normal anxiety was defined as a GAD-7 score <10, while moderate-to-vigorous anxiety was defined as a GAD-7 score ≥10 [29]. In this study, a score greater or equal to 10 was defined as with anxiety symptoms.

### Sexual Relationship Characteristics

#### Emotional Connection With a Sexual Partner During Sex

The participants were asked, “How often would you say you feel emotionally close to your partner when you have sex together? (options: always, most of the time, sometimes, not very often, hardly ever).” The participants who selected the options of “always” and “most of the time” were defined as having a “good emotional connection with a sexual partner during sex;” otherwise, they were defined as having a “poor emotional connection with a sexual partner during sex.”

#### Relationship With Sex Partners

Regarding relationships with sex partners, the participants were asked, “How do you evaluate the relationship with your recent sex partner? Please assign a rating to the quality of your partnership with them.” The rating scale ranges from 1 to 7, with 1 indicating “very good” and 7 representing “very poor.” We categorized the responses into 2 categories for analysis: good relationship with a sex partner (scores 1‐4) and poor relationship with a sex partner (scores 5‐7).

### Sexual Satisfaction

Sexual satisfaction was measured on a 5-point Likert scale, with responses ranging from 1 to 5 (strongly agree, agree, medium, disagree, and strongly disagree). In this study, we reclassified scores of 1‐3 as sexual satisfaction and scores of 4‐5 as sexual dissatisfaction [30].

### Statistical Analyses

Descriptive analyses were conducted to characterize the study sample, including presenting percentages, means, and SD. The *χ*^2^ test was used to compare the proportions of characteristics between the sex groups.

For the multivariable logistic regression analysis, collinearity diagnostics were initially performed for all potential variables (MultimediaAppendices 1  2). Subsequently, multivariate logistic regression analysis was carried out for noncollinear variables. The multivariable logistic regression models selected variables using a stepwise method based on the Akaike Information Criterion (AIC). The stepwise regression method combines forward selection and backward elimination approaches, adding and removing predictors in the model-building process. This approach effectively minimizes the inclusion of covariates, thereby enhancing the robustness of the analysis. Finally, the model with the minimum AIC was adopted (men: 1005.929; women: 582.316). Adjusted odds ratios (aORs) and their corresponding 95% CIs were estimated.

All statistical analyses were performed using R software version 4.2.3 (R Project). The Stats package (version 4.2.2) was used to build the stepwise multivariable logistic regression models. In addition, the figures were generated using the ggplot2 package (version 3.4.3) and forestmodel package (version 0.6.2) from CRAN.

## Results

### Demographic and Health Characteristics of the Participants

As shown in Table 1, 1317 older adults were included in this analysis. The average age was 64 years (SD 8.4 years, ranging from 50 to 86 years). Over half of the participants resided in rural areas (men: 53.1% [447/842], women: 50.9% [242/475]), and the majority reported 7‐12 years of education (junior or senior high school; men: 70.0% [589/842], women: 59.6% [283/475]). In addition, a significant proportion of participants reported infrequent engagement in physical exercise (men: 54.8% [461/842], women: 48.2% [229/475]). Regarding physical health, more than half of the participants did not frequently experience fatigue (men: 85% [716/842], women: 74.9% [356/475]) and did not have chronic diseases (men: 55.2% [465/842], women: 62.9% [299/475]). Regarding sexual relationship characteristics, the majority of male participants reported sexual satisfaction (479/842, 56.9%) and a good relationship with their sex partner (615/842, 73.0%). In comparison, women reported slightly lower rates of sexual satisfaction (228/475, 48.0%) and a good relationship with their sex partner (306/475, 64.4%). Furthermore, less than 5.1% (24/475) of both men and women reported symptoms of anxiety and depression.

**Table 1.** Demographic, lifestyle, health, and sexual relationship characteristics among older adults aged more than 50 years in China (stratified by sex).

Characteristics	Men				Women			
				*P* values				*P* values
At least one of the sexual response problems, n (%)	All	Yes	No		All	Yes	No	
	842 (100.0)	363 (43.1)	479 (56.9)	N/A^a^	475 (100.0)	247 (52.0)	228 (48.0)	N/A
Demographic characteristics								
Living area				<.001				<.001
Rural	447 (53.1)	132 (29.5)	315 (70.5)		242 (50.9)	89 (36.8)	153 (63.2)	
Urban	395 (46.9)	231 (58.5)	164 (41.5)		233 (49.1)	158 (67.8)	75 (32.2)	
Age (years)				.04				.13
50‐59	440 (52.3)	175 (39.8)	265 (60.2)		272 (57.3)	137 (50.4)	135 (49.6)	
60‐69	300 (35.6)	134 (44.7)	166 (55.3)		156 (32.8)	79 (50.6)	77 (49.4)	
70+	102 (12.1)	54 (52.9)	48 (47.1)		47 (9.9)	31 (66)	16 (34)	
Monthly income (RMB)				.37				.72
≥5000	229 (27.2)	105 (45.9)	124 (54.1)		398 (83.8)	205 (51.5)	193 (48.5)	
<5000	613 (72.8)	258 (42.1)	355 (57.9)		77 (16.2)	42 (54.5)	35 (45.5)	
Education level				.009				.02
≤6	160 (19.0)	75 (46.9)	85 (53.1)		121 (25.5)	51 (42.1)	70 (57.9)	
7‐12	589 (70.0)	236 (40.1)	353 (59.9)		283 (59.6)	152 (53.7)	131 (46.3)	
>12	93 (11.0)	52 (55.9)	41 (44.1)		71 (14.9)	44 (62)	27 (38)	
Lifestyle characteristics								
Physical exercise				.81				.001
Often	381 (45.2)	162 (42.5)	219 (57.5)		246 (51.8)	109 (44.3)	137 (55.7)	.
Not Often	461 (54.8)	201 (43.6)	260 (56.4)		229 (48.2)	138 (60.3)	91 (39.7)	
Seeking professional help for sex life				.001				.96
Yes	266 (31.6)	138 (51.9)	128 (48.1)		138 (29.1)	71 (51.4)	67 (48.6)	
No	576 (68.4)	225 (39.1)	351 (60.9)		337 (70.9)	176 (52.2)	161 (47.8)	
Physical health characteristics								
Frequently experienced fatigue				<.001				.07
Often	126 (15.0)	86 (68.3)	40 (31.7)		119 (25.1)	71 (59.7)	48 (40.3)	.07
Not often	716 (85.0)	277 (38.7)	439 (61.3)		356 (74.9)	176 (49.4)	180 (50.6)	
BMI				.001				<.001
Normal	434 (51.5)	162 (37.3)	272 (62.7)		230 (48.4)	95 (41.3)	135 (58.7)	
Abnormal	408 (48.5)	201 (49.3)	207 (50.7)		245 (51.6)	152 (62)	93 (38)	
Sleep quality				<.001				.35
Good	630 (74.8)	232 (36.8)	398 (63.2)		314 (66.1)	158 (50.3)	156 (49.7)	
Poor	212 (25.2)	131 (61.8)	81 (38.2)		161 (33.9)	89 (55.3)	72 (44.7)	
Chronic disease				.10				.06
Yes	377 (44.8)	163(43.2)	214 (56.8)		176 (37.1)	81 (46)	95 (54)	
No	465 (55.2)	200 (43)	265 (57)		299 (62.9)	166 (55.5)	133 (44.5)	
Mental health characteristics								
Anxiety symptoms				.002				.34
Yes	17 (2.0)	14 (82.4)	3 (17.6)		20 (4.2)	13 (65)	7 (35)	
No	825 (98.0)	349 (42.3)	476 (57.7)		455 (95.8)	234 (51.4)	221 (48.6)	
Depressive symptoms				<.001				.012
Yes	37 (4.4)	30 (81.1)	7 (18.9)		24 (5.1)	19 (79.2)	5 (20.8)	
No	805 (95.6)	333 (41.4)	472 (58.6)		451 (94.9)	228 (50.6)	223 (49.4)	
Sexual relationship characteristics								
Emotional connection with a sexual partner during sex				<.001				.004
Yes	573 (68.1)	223 (38.9)	350 (61.1)		294 (61.9)	144 (49)	150 (51)	
No	269 (31.9)	140 (52.0)	129 (48.0)		181 (38.1)	103 (56.9)	78 (43.1)	
Relationship with a sex partner				.002				.005
Good	615 (73.0)	245 (39.8)	370 (60.2)		306 (64.4)	144 (47.1)	162 (52.9)	
Poor	227 (27.0)	118 (52)	109 (48)		169 (35.6)	103 (60.9)	66 (39.1)	
Sexual satisfaction^b^				<.001				<.001
Yes	479 (56.9)	0 (0)	479 (100)		228 (48.0)	0 (0)	228 (100)	
No	363 (43.1)	363 (100)	0 (0)		247 (52.0)	247 (100)	0 (0)	

a N/A: not applicable

b Sexual response problems include lacked interest in having sex, lacked enjoyment in sex, feeling anxiety during sex, feeling physical pain as a result of sex, feeling no excitement or arousal during sex, difficulty in reaching climax, reaching climax more quickly than desired, trouble getting or keeping an erection(men) or uncomfortable dry vagina(women).

### Prevalence of Sexual Response Problems

**Figure 1.**
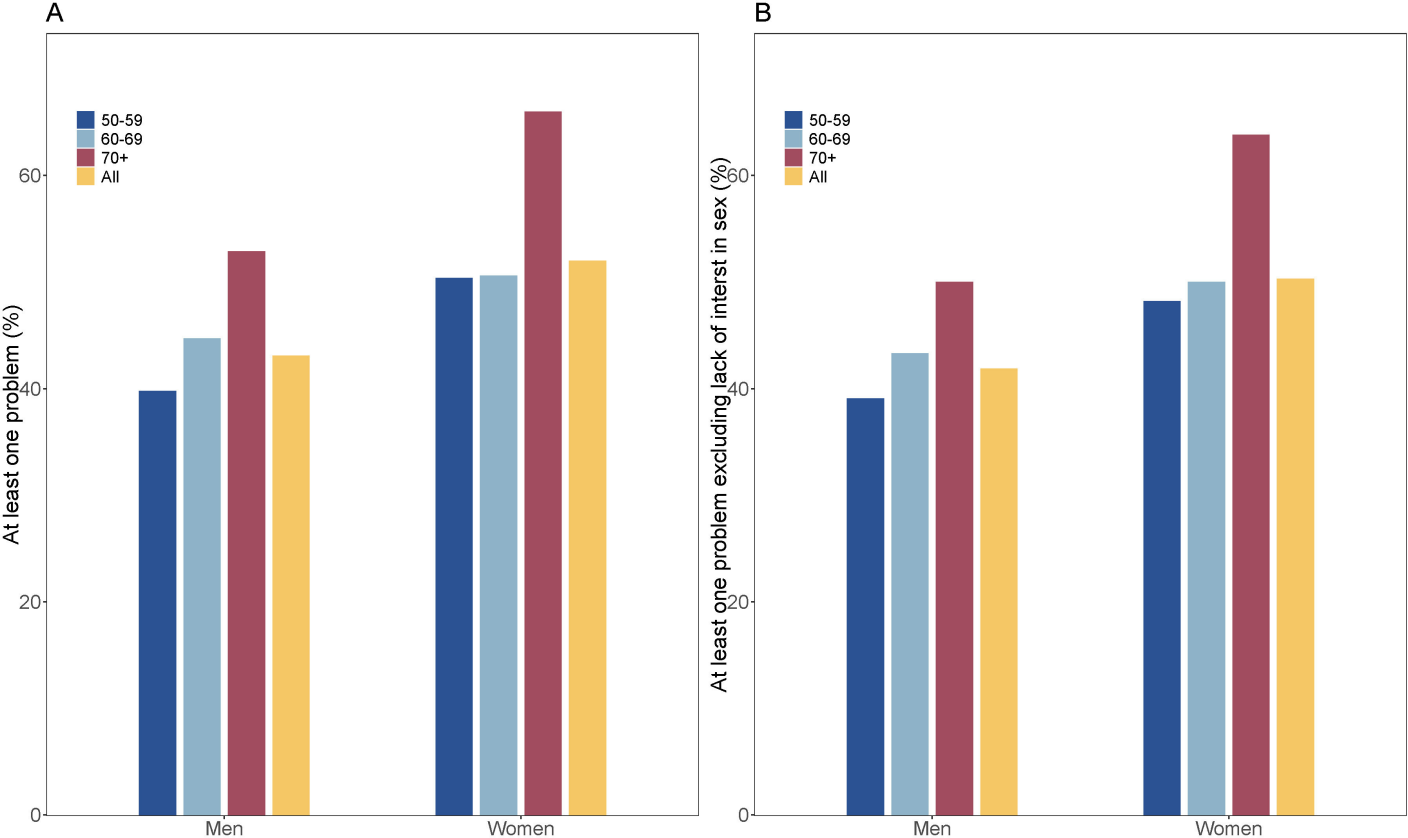
Prevalence of sexual response problems among older adults aged 50+ years**.** (**A**) At least one of sexual response problems among men and women; (**B**) At least one of sexual response problems excluding lack of interest in sex among men and women.

The prevalence of at least one sexual response problem (including or excluding lack of interest in sex) is shown in Figure 1 and Table 2. In total, 610 out of 1317 participants had sexual response problems, with an overall prevalence of sexual response problems of 46.3% (610/1317). There was a significant difference in the prevalence of the reported at least one sexual response problem between women and men, with being significantly higher in women than in men (52.0% [247/475] vs 43.1% [363/842], *χ*^2^_1_=9.6, *P*=.002). The prevalence of at least one sexual response problem increased with age among men (The Cochran-Armitage Trend Test, Z=−2.476, *P* for trend=.01). However, this trend was only observed in the age group between the older than 70 years age group and the other 2 age groups among men. There was no significant difference in the prevalence of sexual response problems between the 60‐69 years age group and the 50‐59 age group among women.

**Table 2.** The prevalence of sex response problems among men and women by age group.

Age group	Men	Women	Chi-square (df)	*P* values
At least one sex response problem				
50‐59	175 (39.8)	137 (50.4)	7.24 (1)	.007
60‐69	134 (44.7)	79 (50.6)	1.24 (1)	.27
Older than 70 years	54 (52.9)	31 (66.0)	1.73 (1)	.19
All age groups	363 (43.1)	247 (52.0)	9.65 (1)	.002
At least one sex response problem excluding lack of interest in sex				
50‐59 years	172 (39.1)	131 (48.2)	5.29 (1)	.02
60‐69 years	130 (43.3)	78 (50.0)	1.58 (1)	.21
Older than 70 years	51 (50)	30 (63.8)	1.95 (1)	.16
All age groups	353 (41.9)	239 (50.3)	8.31 (1)	.004

### Correlates of Sexual Response Problems

**Figure 2.**
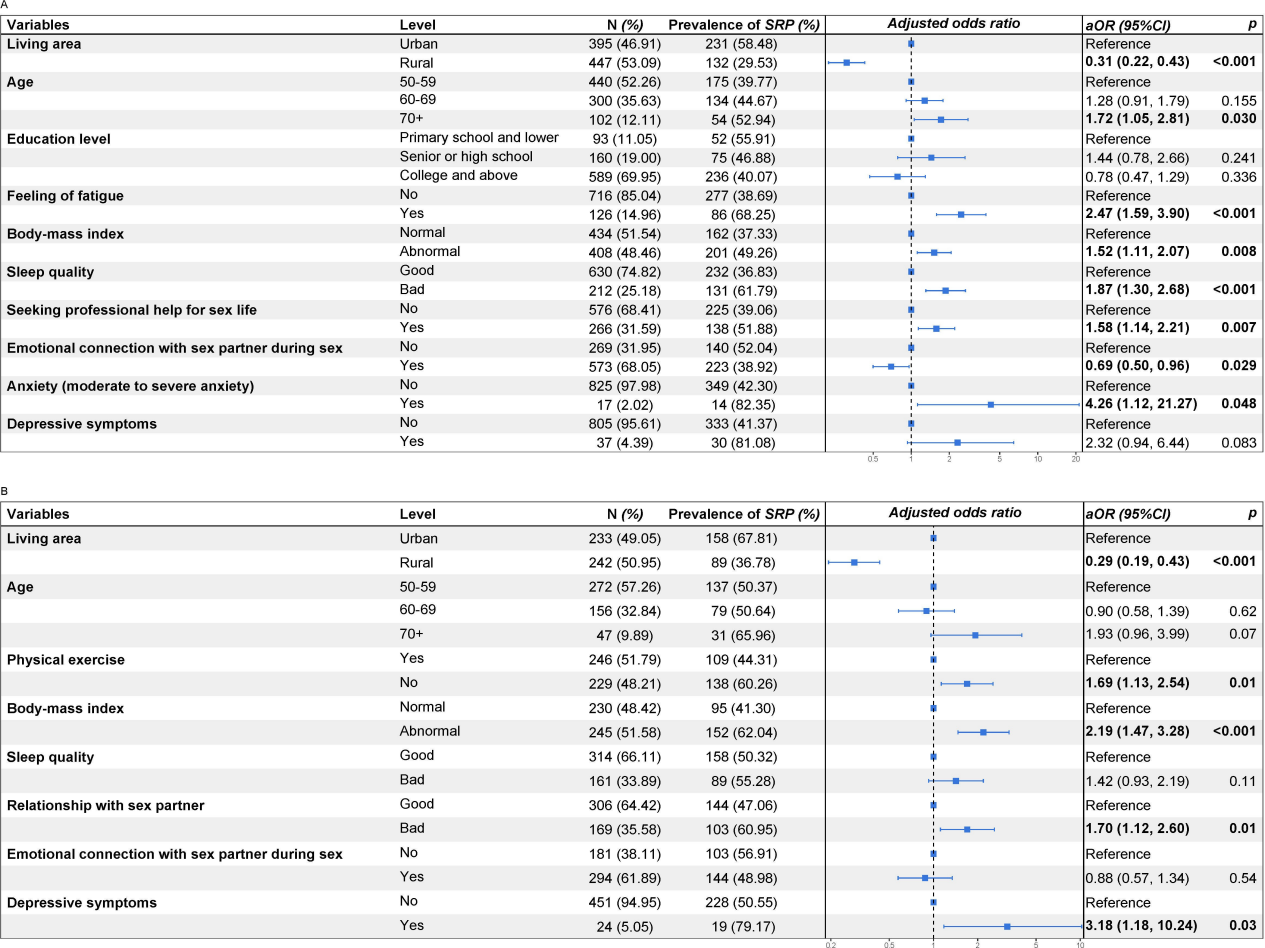
Correlates of sexual response problems among older adults aged 50+ years**.** (**A**) Correlates of sexual response problems among men aged 50+ years; the multivariable logistic regression analysis was adjusted by living area, age, educational level, feeling of fatigue, body mass index, sleeping quality, seeking professional help for sex life, emotional connection with sexual partner during sex, anxiety, depressive symptoms. (**B**) Correlates of sexual response problems among women aged 50+ years; the logistic regression model was adjusted by age, living area, physical exercise, body-mass index, sleeping quality, sexual relationship with partner, emotional connection with sexual partner during sex, depressive symptoms. SRP: Sexual Response Problem; aOR: adjusted odds ratio; CI: confidence interval.

The results of multivariable logistic regression analysis stratified by sex, which was presented in Figure 2, revealed several significant associations with reporting at least one sexual response problem.

For older men, residing in rural areas (aOR 0.31, 95% CI 0.22‐0.43) and maintaining an emotional connection with a sex partner during sexual intercourse (aOR 0.69, 95% CI 0.50‐0.96) were associated with a reduced likelihood of reporting sexual response problems. In contrast, being 70 years or older (aOR 1.72, 95% CI 1.05‐2.81), frequently experiencing fatigue (aOR 2.47, 95% CI 1.59‐3.90), and poor sleeping quality (aOR 1.87, 95% CI 1.30‐2.68) were associated with higher odds of reporting sexual response problems. In addition, moderate to severe anxiety symptoms (aOR 4.26, 95% CI 1.12‐21.27), abnormal BMI (aOR 1.52, 95% CI 1.11‐2.07), and seeking professional help for sex issues (aOR 1.58, 95% CI 1.14‐2.21) were positively associated with reporting sexual response problems among older men.

Older women residing in rural areas (aOR 0.29, 95% CI 0.19‐0.43) were less likely to report sexual response problems. Conversely, factors such as infrequent engagement in physical exercise (aOR 1.69, 95% CI 1.13‐2.54), depressive symptoms (aOR 3.18, 95% CI 1.18‐10.24), having an abnormal BMI (aOR 2.19, 95% CI 1.47‐3.28), and being in a poor sex partner relationship (aOR 1.70, 95% CI 1.12‐2.60) were associated with higher odds of reporting sexual response problems.

## Discussion

### Principal Results

Our study demonstrated a high prevalence of sexual response problems among older adults, with variations noted between men and women. In older men, sexual response problems correlate with advancing age. Our findings also linked sexual response problems with adverse physical health outcomes such as frequent experiencing fatigue, poor sleep quality, and abnormal BMI. In addition, we observed strong associations between sexual response problems and mental health issues, including anxiety and depressive symptoms. Moreover, poor sex partner relationships, sexual dissatisfaction, and lack of emotional connection during sex were also associated with sexual response problems. These findings significantly contribute to the existing literature, underscoring the importance of addressing sexual response problems within the domain of sexual health and enhancing our understanding of these issues among older adults.

### Comparison With Previous Work

In this study, women reported a higher prevalence of sexual response problems compared to men, consistent with findings from other countries [12-14]. This discrepancy may be due to several factors, including physiological alterations, psychological elements, and sociocultural dynamics. With advancing age, women experience a significant decline in estrogen levels, especially after menopause [31]. Furthermore, older women are subject to various age-related psychological changes, such as concerns about body image, fears about aging, and self-consciousness about their sexual lives, which may lead to decreased sexual desire or arousal issues. In Chinese culture, the sexual lives of older adults are frequently neglected or deemed inappropriate, which may influence the perceptions and expectations of sex among older women, making them feel ashamed or uncomfortable in their sexual lives. This sex difference highlights the need for targeted interventions for women’s sexual health. These interventions should address the multifaceted nature of sexual health in older women, combining physiological, psychological, and sociocultural interventions.

In this study, adverse physical health, such as abnormal BMI, frequent fatigue, and poor sleep quality, played a crucial role in sexual response problems. These findings were identified by evidence from high-income countries, which linked poor physical health to sexual response problems [32-34]. Obesity, characterized by an abnormal BMI, detrimentally impacts the reproductive system and sexual function [35-37]. Both men and women affected by obesity face a heightened risk of fertility challenges and sexual dysfunction [38]. Weight loss can reduce fatty tissue and diminish aromatase activity, leading to a relative increase in testosterone levels [39], which enhances sexual function in men. Besides, weight loss tends to improve sexual functioning for women and men [40]. Obesity significantly affects the hypothalamic-pituitary-gonadal axis in men, leading to diminished libido and erectile dysfunction [41]. The previous review highlighted that excess body weight negatively affected hormones contributing to sexual behavior, noting that adipose tissue facilitates the conversion of androgens to estrogens, further impacting sexual function [42]. Given the inverse association between body mass and sexual response problems, it is recommended that clinicians, both in general practice and in weight loss programs, should more fully address sexual response problems.

Poor sleep quality was associated with sexual response problems in previous studies [43,44], which was also demonstrated by this study. Poor sleep is closely linked to sexual dysfunction due to several physiological and psychological factors. Inadequate sleep can disrupt hormone production, notably reducing testosterone levels, which are essential for sexual desire and performance. Studies have shown that sleep deprivation can decrease testosterone production, leading to impaired sexual activity [45]. In addition, sleep disorders often contribute to stress, anxiety, and depression, all of which can diminish libido and sexual satisfaction [46]. Furthermore, chronic stress and anxiety can lead to sleep problems, which in turn may cause erectile dysfunction [47]. Sleep is fundamental to health, and its bidirectional relationship with sexual response difficulties necessitates that clinicians conduct thorough assessments to identify underlying causes of poor sleep, encourage patient-partner communication to alleviate psychological burdens, promote regular physical activity to enhance sleep quality, and recommend professional sleep therapy to improve physiological function when necessary.

Our study showed that lack of physical exercise was correlated with the occurrence of sex response problems among older women. Regular physical activity is a healthy practice that can mitigate the risk of sexual response problems [36,48]. These associations between sexual response problems and physical health underscore the importance of prioritizing sexual function within sexual health. Physical exercise and high-quality sleep are recommended, along with other lifestyle guidance, to improve sexual functioning in both men and women and to improve health across a range of domains.

Psychosocial factors, including symptoms of anxiety and depression, exhibited the strongest association with sexual response problems, as evidenced by findings from the Global Study of Sexual Attitudes and Behaviours (GSSAB) [49]. Other studies also linked the associations between mental health and sexual response problems, which highlighted that men with anxiety disorders are at a higher risk of developing erectile dysfunction [50,51]. Moreover, the physiological responses triggered by heightened anxiety levels may contribute to disruptions in sexual function [52]. This finding will capture the attention of practitioners exploring the causes and treatment of sexual problems in patients. In clinical practice, there should be heightened efforts to address sexual problems within integrated services of mental health and sexual health counseling, as well as in primary and secondary care.

Our findings underscored the importance of sex-partner relationships in the context of sexual response problems. Specifically, men who maintained an emotional connection with their sex partners during sex were less likely to report sex response problems, while women experiencing poor relationships with sex partners were more likely to encounter sexual response problems in older women. This underscores the role of brief emotional interactions during sexual activity in men’s adaptation to sexual response problems. In contrast, long-term sexual relationships appear to be more influential in women’s adjustment to sexual response problems. Existing studies have consistently underscored the significance of partner relationships for sexual response problems in women [53], emphasizing the impact of daily intimacies in relationships [9]. In contrast, emotional intimacy is presumed to play a significant role in maintaining sexual desire and partnered sexual activity for men [54,55]. In addition, emotional connection with a partner may foster open communication, trust, and mutual understanding, all essential to a satisfying sexual relationship. The variation in sexual response problems concerning sex partner relationships among older men and women underscores the importance of physicians considering sex differences when diagnosing and treating patients with sexual response problems. In future studies, the sex partner should be included and involved in the evaluation and management to achieve a better intimate relationship in an established couple and avoid sexual response problems.

### Limitations

Our study has several limitations that should be considered. First, as a cross-sectional study, it cannot establish temporal order and causal direction. Second, the reliance on self-reporting in the questionnaires, especially for sensitive issues, introduced the possibility of recall biases. Third, the sexual response problems were reported by the participants experiencing them, and the differences in the sensitivity and understanding of the same sexual problem may exist among participants, potentially influenced by factors such as education, age, or living area. Finally, some analyses were based on small cell sizes, particularly for variables like anxiety and depressive symptoms, which may result in unstable estimates.

### Conclusion

This study showed a substantial prevalence of sexual response problems among older adults, with women experiencing these problems more often than men. The study identified adverse physical health, poor mental health, and poor relationships with sex partners as factors contributing to increased sexual response problems among older adults. To address these concerns, health care professionals can implement interventions for older adults experiencing sexual response problems, such as enhancing physical health, supporting mental health, improving intimate relationships, and providing educational and cognitive-behavioral interventions. These insights drawn from the latest and representative SWELL study data enhance our understanding of sexual response problems among older adults and have the potential to promote overall health and well-being among the aging population in China.

## Supplementary material

10.2196/66772 Multimedia Appendix 1 Collinearity diagnostics of all potential variables for the multivariable logistic regression analysis (Men). Note that no value was more than 0.3, suggesting no collinearity between variables.

10.2196/66772 Multimedia Appendix 2 Collinearity diagnostics of all potential variables for the multivariable logistic regression analysis (Women). Note that no value was more than 0.3, suggesting no collinearity between variables.

## Abbreviations

AIC: Akaike Information Criterion
aORs: adjusted odds ratios
GAD-7: generalized anxiety disorder-7
GSSAB: Global Study of Sexual Attitudes and Behaviours
PHQ-9: Patient Health Questionnaire
SQS: sleep quality scale
SRPs: Sexual response problems
SWELL: Sexual Well-being
