# Supplementary figures and images for: Sexual Response Problems and Their Correlates Among Older Adults From the Sexual Well-Being (SWELL) Study in China: Multicenter Cross-Sectional Study
Source: JMIR Aging. 2025 May 1;8:e66772. doi: 10.2196/66772 (PMC12061351; doi:10.2196/66772)

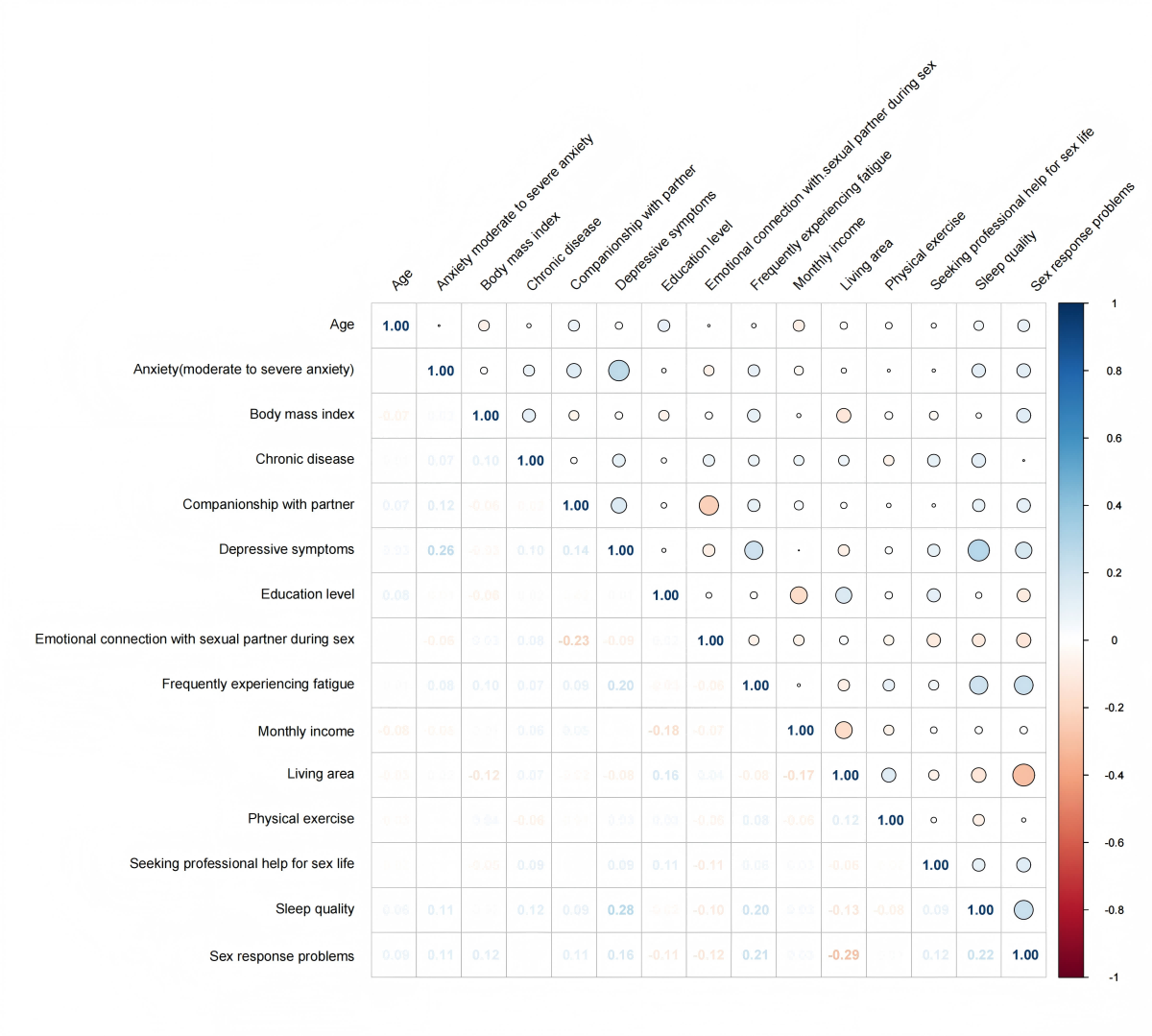

Supplement: Multimedia Appendix 1 [file aging-v8-e66772-s001.png]

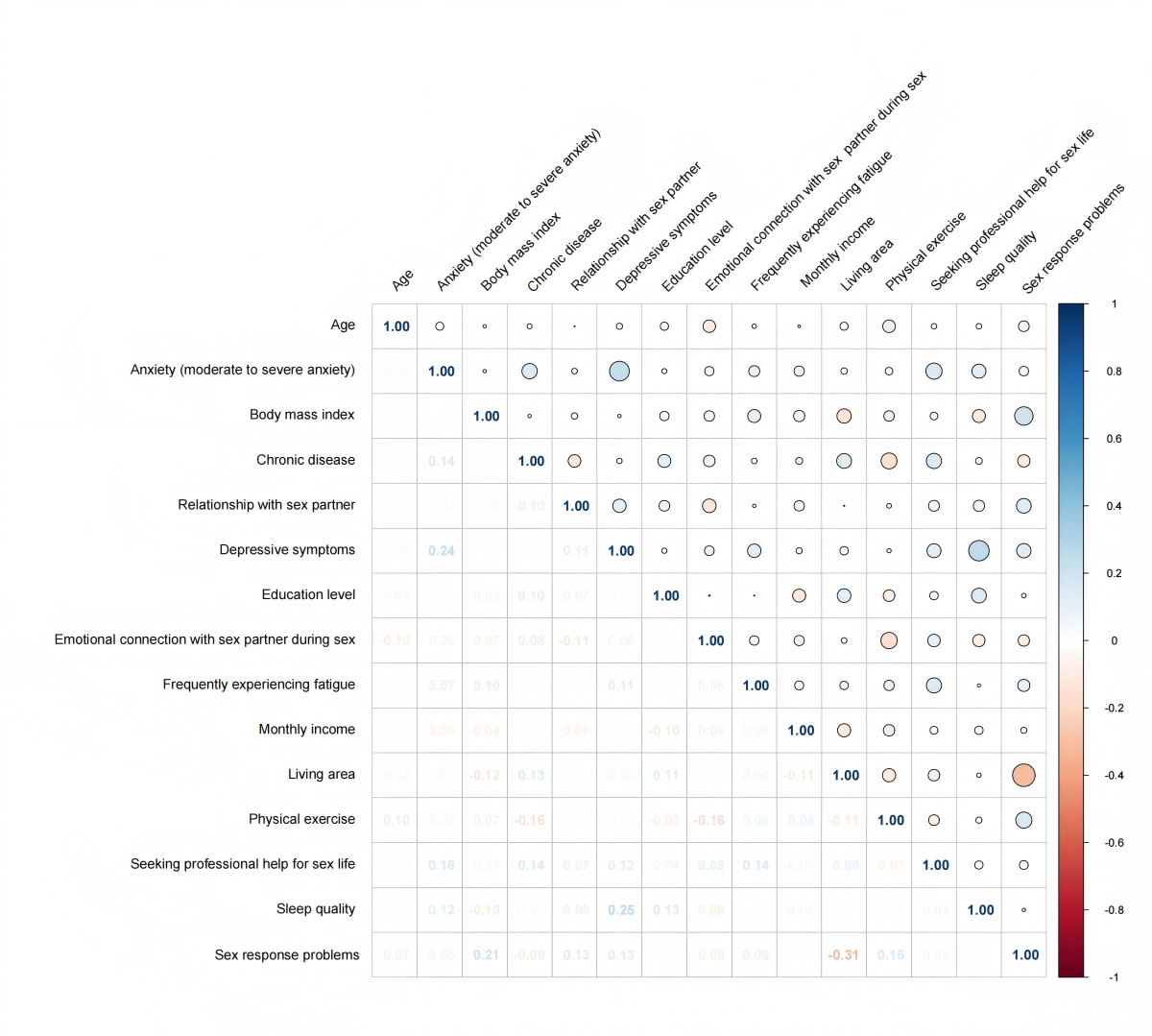

Supplement: Multimedia Appendix 2 [file aging-v8-e66772-s002.png]
